# Guidelines for replacement of a balloon gastrostomy tube in infants and pediatric patients: The American Society for Parenteral and Enteral Nutrition

**DOI:** 10.1002/jpen.70080

**Published:** 2026-03-26

**Authors:** Beth Lyman, Loren Berman, Kathleen Carr, Cailin Frank, Peggi Guenter, Rachel Kassel, Janet Kimble, Carol McGinnis, Silvana Oppedisano, Elizabeth A. Paton, Gina Rempel, Derek S. Wakeman, David D. Church, Jacob T. Mey, Sarah Peterson, Liam McKeever

**Affiliations:** ^1^ Nutrition, Support Nurse Consultant Smithville Missouri USA; ^2^ Nemours Children's Health Wilmington Delaware USA; ^3^ Boston Children's Hospital Boston Massachusetts USA; ^4^ Le Bonheur Children's Hospital University of Tennessee Health Science Center Memphis Tennessee USA; ^5^ American Society for Parenteral and Enteral Nutrition Silver Spring Maryland USA; ^6^ Department of Pediatrics, Division of Pediatric Gastroenterology, Hepatology, and Nutrition University of Alabama at Birmingham Heersink School of Medicine Birmingham Alabama USA; ^7^ Cedars‐Sinai Medical Center Los Angeles California USA; ^8^ Sanford USD Medical Center Sioux Falls South Dakota USA; ^9^ Division of Paediatric Medicine, G‐Tube Feeding Program The Hospital for Sick Children Toronto Ontario Canada; ^10^ Department of Acute and Tertiary Care, College of Nursing, Le Bonheur Children's Hospital University of Tennessee Health Science Center Memphis Tennessee USA; ^11^ Department of Pediatrics and Child Health Nutrition Support and Complex Care, HSC Winnipeg, Shared Health Winnipeg Manitoba Canada; ^12^ University of Rochester Strong Memorial Hospital Rochester New York USA; ^13^ Department of Geriatrics University of Arkansas for Medical Sciences Little Rock Arkansas USA; ^14^ Pennington Biomedical Research Center Baton Rouge Louisiana USA; ^15^ Department of Clinical Nutrition Rush University Medical Center Chicago Illinois USA; ^16^ Department of Clinical Nutrition, College of Health Sciences Rush University Chicago Illinois USA

**Keywords:** balloon gastrostomy tube, care bundles, clinician education, pediatrics, placement verification, practice guideline, radiologic contrast study, tube dislodgement, tube replacement, ultrasound

## Abstract

**Background:**

Balloon gastrostomy tube (BGT) placements are increasing due to their ability to facilitate care of medically complex children. Management for routine and nonroutine tube replacement, including verification of proper replacement, lacks standardization, varying widely among different institutions and settings. Currently, no overall standard of care exists for placement verification following BGT replacement, which can occur anywhere. This guideline provides recommendations on topics surrounding best practices, timing, and education confirming newly replaced BGTs.

**Methods:**

An interdisciplinary team developed key questions and performed a systematic search of the PubMed/MEDLINE, EMBASE, CINAHL, and Cochrane Central databases from January 2008 to October 2025. Recommendations were developed for questions concerning timing of routine BGT replacement, weighing the impact of comorbidities and extenuating circumstances, standard and additional methods to confirm BGT replacement position, confirmation of placement when the BGT is traumatically or accidentally dislodged, and how care bundles and clinician education around BGT replacement impact patient outcomes.

**Results:**

Three studies were included (one validation and two pre‐post studies). Recommendations were made for when additional measures beyond aspiration of gastric contents (e.g., radiographic contrast study) are indicated, use of ultrasound in lieu of a contrast study, what care bundle components improve patient outcomes, and what topics should be covered in clinician education for BGT replacement confirmation.

**Conclusion:**

This guideline provides placement confirmation guidance for clinicians who perform routine and nonroutine BGT replacements, identifies areas for research, and suggests the use of care bundles to standardize care of this population. This paper was approved by the ASPEN Board of Directors.

## PURPOSE

Rates of gastrostomy tube (GT) placement are rising, due in part to refined placement techniques and an increased appreciation for the role of nutrition support in growth.^1^, ^2^, ^3^ A recent publication of aggregated data from 157 children's hospitals documented that 5.3% of all surgical procedures were for GT placement.^4^ In addition to surgeons, interventional radiologists and gastroenterologists also place and manage GTs, along with experienced nurses and advanced practice professionals. Most pediatric patients have low‐profile balloon gastrostomy (LPBG) tubes, as patients and caregivers appreciate the LPBG aesthetic and ease of use. These devices sit at skin level, may be readily concealed, provide limited interference with clothing, and are thought to have fewer adverse events in terms of accidental dislodgement and leakage than percutaneous tubes.^5^

Balloon gastrostomy tubes (BGTs) require replacement for both routine wear and tear and unexpected dislodgement. Indeed, the study from 157 children's hospitals reported dislodgement rates of 5.2% and 5.5% for 0–30 days and 31–60 days post placement, respectively.^4^ Management for routine and nonroutine tube replacement, including the verification of proper replacement, lack standardization and vary widely among different institutions and settings. Many institutions have developed guidance for decisions regarding BGT replacement. Currently, however, no overall standard of care exists for placement verification following BGT replacement. Replacement may occur in pediatric inpatient units, emergency departments (ED), outpatient clinics, residential pediatric care facilities, and outside clinical environments (i.e., at the child's home or school). There are no agreed‐upon standards for when the initial tube change should be performed or for the subsequent frequency regarding routine BGT exchange or nonroutine replacement management, such as when dilation of the stoma is required.

While trained caregivers can replace the BGT at home, a lack of replacement kits, difficulty in replacement, or discomfort with the procedure may prompt them to come to an ED for assistance.^6^ Inappropriate use of ED services could be averted with comprehensive education and outpatient support.^7^ When a GT is inadvertently displaced, the patient and caregiver often come to the ED, as timely replacement of a displaced GT is required to prevent stomal stenosis.^8^ Although commonly treated in the ED, emergency medical care for displaced GTs not only diverts ED staff but also inconsistently addresses replacement, confirmation of placement, and documentation, and reinforces ED use rather than access to healthcare through specialty clinics or advanced practice providers.^9^

Lack of evidence‐based practice standards for BGT replacement may result in misplaced tubes or overuse of radiologic contrast studies. Feeding via misplaced tubes carries serious consequences (e.g., ED visits, hospital readmissions, additional surgical interventions).^10^ Tract disruption is an adverse consequence of GT replacement,^6^ which may subsequently lead to dislodgment, leakage of gastric contents, infection, development or worsening of granulation tissue, or peritonitis.^11^ Therefore, verification of appropriate placement prior to tube use is essential to detect potential misplacement or other adverse events.

In 2012 the American Society for Parenteral and Enteral Nutrition (ASPEN) convened the workgroup *New Opportunities of Verification of Enteral Tube Location* (*NOVEL*) as an interorganizational, interdisciplinary, multinational assemblage including a parent member to address nasogastric tube (NGT) misplacement issues. After intense and thorough evaluation, this group identified standards of practice that are being disseminated and implemented around the world to enhance the safety of practice.^12^ After that work and upon the suggestion of the ASPEN Pediatric Section, a multiorganizational, interdisciplinary workgroup was convened to address BGT replacement verification and develop clinical guidelines to enhance the safety of replacing BGT in pediatric patients.

The objective of the current guideline is to provide guidance for both the routine and nonroutine (i.e., emergent) replacement of a BGT in infants and pediatric patients via a systematic review, grading of the literature, and a modified Delphi to assess agreement for the formulated recommendations. A complete list of the Guideline Recommendations is presented in Table 1.

**Table 1:** Guideline questions, evidence grades, recommendations.

**1.**	**In infants and pediatric patients receiving routine replacement of a BGT, does confirming gastric tube placement via gastric aspiration with pH versus gastric aspiration without pH result in fewer adverse events?**
	*Recommendation*: In infants or pediatric patients with a BGT requiring routine replacement, we recommend that the gastric fluid be aspirated to confirm placement. Gastric pH measurement may not be necessary as a first‐line method.
	*Certainty of evidence*: Very Low/Expert Opinion
	*Strength*: Strong
**2.**	**In infants and pediatric patients undergoing the initial replacement of a BGT, does waiting more time versus less time from initial placement result in fewer negative clinical outcomes?**
	*Recommendation*: In infants or pediatric patients with a BGT, we recommend waiting 6–12 weeks after BGT placement for the initial replacement.
	*Certainty of evidence*: Very Low/Expert Opinion
	*Strength*: Strong
**3.**	**In infants and pediatric patients receiving a routine replacement of a BGT and in whom gastric aspirate is not attainable, does confirming gastric tube placement via ultrasound (US) versus radiologic contrast study result in fewer negative clinical outcomes?**
	*Recommendation*: In infant or pediatric patients with a BGT, when gastric aspirate is not attainable, we suggest using US, when feasible, to verify gastric position post replacement. Failure to obtain a verification of correct BGT replacement using US requires a radiologic contrast study to confirm that the balloon is correctly placed within the stomach.
	*Quality of evidence*: Very Low/Expert Opinion
	*Strength*: Weak
**4.**	**In the infant and pediatric patient receiving routine replacement of a BGT who also has a peritoneal dialysis (PD) catheter or ventriculoperitoneal (VP) shunt, does confirming gastric tube placement via aspiration (with or without pH) versus radiologic contrast study result in fewer negative clinical outcomes?**
**5.**	**In the infant and pediatric patients receiving routine replacement of a BGT who also has a peritoneal dialysis (PD) catheter or ventriculoperitoneal (VP) shunt, does waiting more time versus less time post initial tube placement result in fewer negative clinical outcomes?**
	*Recommendation*: In infants or pediatric patients with a VP shunt requiring routine replacement of a BGT, we recommend aspiration of gastric fluid as the first‐line method to confirm gastric placement.
	In infants or pediatric patients with a PD catheter requiring routine replacement of a BGT where there is no fluid leakage onto the abdomen from around the stoma, we recommend aspiration of gastric fluid as the first line method to confirm gastric placement.
	In infants or pediatric patients with a BGT and PD catheter or VP shunt receiving routine initial replacement of the BGT, we suggest waiting more time—that is, closer to the previously mentioned 12 weeks post placement if peristomal fluid is leaking onto the abdomen. If feasible, waiting until the peristomal area is without fluid leakage is ideal. Otherwise, with fluid leakage, we recommend following the institutional policy for when initial routine BGT replacement is performed.
	If replacement is indicated or necessary, we suggest observing the appearance of gastric aspirate before and after BGT replacement to compare for similarity of appearance. Alternatively, we recommend instilling a small amount of a colored beverage into the existing BGT prior to removal to compare fluid aspirated from the newly placed BGT for similarity of appearance. If indicated, or previous methods have been inconclusive, we recommend obtaining a radiologic contrast study to confirm placement of the newly replaced BGT. For institutions with proficiency in using US for BGT replacement verification, this would be preferred over radiologic contrast study.
	*Quality of evidence*: Very Low/Expert Opinion
	*Strength*: Strong
**6.**	**In infants or pediatric patients receiving routine initial replacement of a BGT, where the patient has conditions that could adversely affect stoma maturation such as kidney disease requiring a peritoneal dialysis catheter (PD), neurologic condition requiring a ventriculoperitoneal (VP) shunt, an oncologic condition requiring chemotherapy, diabetes, or a history of chronic steroid use, does waiting more time versus less time post initial placement result in fewer negative clinical outcomes?**
	*Recommendation*: In infants and pediatric patients who have both a BGT that requires initial replacement and an extenuating comorbidity that may delay wound healing and, therefore, tract maturation, we suggest waiting longer—that is, closer to 12 weeks post placement—to perform the initial BGT replacement. We also suggest not changing the BGT if there is peristomal drainage such as fluid or blood, if possible.
	*Certainty of evidence*: Very Low/Expert Opinion
	*Strength*: Weak
**7.**	**In infants and pediatric patients receiving a routine replacement of the initial BGT, does the use of a care bundle compared to the nonuse of a care bundle result in fewer negative clinical outcomes?**
	*Recommendation*: In infants and pediatric patients with a routine BGT replacement, we recommend the use of a care bundle to standardize and manage patient care, including caregiver education post BGT replacement.
	While we acknowledge that each institution will customize specific information provided to caregivers and older children with a newly placed BGT, we recommend that the elements of the care bundle mimic those in the literature. The components that should be included in a care bundle for caregiver education (including the older child with a newly placed BGT) include: who is permitted to perform initial and subsequent routine BGT replacement; the time period between placement and replacement; what education is provided to caregivers; how that education is provided; and how healthcare literacy is addressed. Other components of the care bundle that deal with patient assessment before BGT placement should include: delineation of the process for initial BGT replacement, including pre‐procedure evaluation and determination of the preferred BGT placement method and setting; an algorithm for evaluating patients presenting to a healthcare facility for nonroutine BGT replacement; and complication management with information on whom to call for help when their regular staff resources are not available. These elements provide a comprehensive care bundle that clinicians can use to standardize care of the child who requires a BGT replacement.
	*Certainty of evidence*: Very Low/Expert Opinion
	*Strength*: Strong
**8.**	**In infants and pediatric patients receiving a routine replacement of a BGT, does the use of formal focused clinician education versus no formal focused clinician education result in fewer negative clinical outcomes?**
	*Recommendation*: In infants or pediatric patients with a BGT, we recommend implementation of a staff education program focusing on routine replacement with didactic and supervised components.
	We recommend that the elements of the program should include, at a minimum, patient assessment, BGT stoma site and size assessment, routine replacement, routine verification of the replaced BGT, signs and symptoms for escalation to elicit support from more senior staff or more invasive interventions, replacement evaluation using nonroutine methods, and protection of the BGT from accidental or premature dislodgement. Supervision of staff who are being trained on routine BGT replacement is recommended, but can be institution specific in terms of the number of supervised routine BGT replacements.
	*Quality of evidence*: Very Low/Expert Opinion
	*Strength*: Strong
**9.**	**In infants and pediatric patients with a BGT that inadvertently comes out before the tract is considered established, does confirming placement of the gastric replacement tube via a radiologic contrast study versus aspiration of gastric contents with or without pH result in fewer negative outcomes?**
	*Recommendation*: In infants and pediatric patients with a BGT that becomes dislodged before the tract is considered mature or established, we recommend confirming the placement of the new BGT via a radiologic contrast study. Institutions with capability and proficiency in US might utilize this technology instead of a radiologic contrast study as the first‐line method for confirmation of the newly replaced BGT in this clinical situation, when feasible.
	*Certainty of evidence*: Very Low/Expert Opinion
	*Strength*: Strong
**10.**	**In infants and pediatric patients with a BGT dislodgement (traumatic or accidental) after tract maturation, does confirming placement of the gastric replacement tube via aspiration of gastric contents with or without pH versus a radiologic contrast study result in fewer negative clinical outcomes?**
	*Recommendation*: In infants or pediatric patients with a mature tract where the BGT becomes dislodged accidentally (i.e., tube falls out with balloon deflated) or traumatically (i.e., tube removed with balloon inflated), we recommend aspiration of gastric contents over the use of a radiologic contrast study as a first‐line method of BGT confirmation.
	*Quality of evidence*: Very Low/Expert Opinion
	*Strength*: Strong
**11.**	**In infants and pediatric patients with a BGT that is difficult to replace, does confirming placement of the gastric replacement tube via a radiologic contrast study versus aspiration of gastric contents with or without pH result in fewer negative outcomes?**
	*Recommendation*: In infants or pediatric patients with a mature tract and BGT that was difficult to replace but did not require dilation, we recommend aspiration of gastric fluid with or without pH as the first‐line method to confirm placement of the replaced BGT. If gastric aspirate is unable to be obtained, it may be appropriate to use a radiologic contrast study to confirm placement of the newly placed BGT. Where US of the BGT is an established practice and feasible, it could be used to verify the newly placed BGT instead of a radiologic contrast study, thereby avoiding radiation exposure.
	*Quality of evidence*: Very Low/Expert Opinion
	*Strength*: Strong
**12.**	**In infants and pediatric patients with a BGT that is difficult to replace and requires the use of a dilator, does confirming placement of the gastric replacement tube via radiologic contrast study versus aspiration of gastric contents result in fewer negative outcomes?**
	*Recommendation*: In infants and pediatric patients with a BGT and mature tract that required dilation prior to replacement, we suggest first attempting gastric aspirate and, if there is any concern about placement, obtaining a radiologic contrast study to confirm placement of the newly replaced BGT.
	Where US of the BGT is an established practice and feasible, it could be used to verify the newly placed BGT instead of a radiologic contrast study, thereby avoiding radiation exposure.
	*Quality of evidence*: Very Low/Expert Opinion
	*Strength*: Weak
**13.**	**In infants and pediatric patients with a BGT that inadvertently comes out, traumatically or accidentally, before the tract is considered established, does confirming placement of the gastric replacement tube via the use of US versus a radiologic contrast study result in fewer negative outcomes?**
	*Recommendation*: In infants and pediatric patients with accidental or traumatic BGT dislodgement that occurs prior to when the tract is considered mature, we suggest using US, which is an emerging confirmation technique that may be equivalent to a contrast study for confirming BGT position. If institutionally feasible, US has the advantage of minimizing radiation. If US is not conclusive, a contrast study should be performed.
	*Quality of evidence*: Very Low/Expert Opinion
	*Strength*: Weak
**14.**	**In infants and pediatric patients with a BGT that comes out traumatically or accidentally before the tract is considered mature, does the use of a care bundle by staff compared to nonuse of a care bundle result in fewer negative outcomes?**
	*Recommendation*: In infants and children with a BGT that comes out accidentally or traumatically before the tract is considered mature, we recommend the use of a care bundle to standardize patient management and caregiver education.
	*Quality of evidence*: Very Low/Expert Opinion
	*Strength*: Strong
**15.**	**In infants and pediatric patients with a BGT that accidentally or traumatically comes out before the tract is considered mature, does the use of a formal clinician education program focused on BGT replacement confirmation versus no education program result in fewer negative outcomes?**
	*Recommendation*: In infants and pediatric patients with a BGT that becomes dislodged accidentally or traumatically before the tract is considered mature, we recommend the development and implementation of a formal education program for clinicians who replace and manage these tubes.
	*Quality of evidence*: Very Low/Expert Opinion
	*Strength*: Strong

Abbreviations: BGT, balloon gastrostomy tube; ED, emergency department; LOS, length of stay; PD, peritoneal dialysis; US, ultrasound; VP, ventriculoperitoneal.

Recommendations in this guideline do not constitute medical or other professional advice and should not be taken as such. To the extent that the information published herein may be used to assist in the care of patients, the primary component of quality medical care is the result of the professional judgment of the healthcare professionals providing care. The information presented here is not a substitute or replacement for the exercise of professional judgment by healthcare professionals; rather, it is intended to supplement professional training and judgment. Circumstances and patient specifics in clinical settings may require actions different from those recommended in this document; in those cases, the judgment of the treating professionals should prevail. Use of this information does not in any way guarantee any specific benefit in outcome or survival. This paper was approved by the ASPEN Board of Directors.

### Target population

The target population for this guideline is pediatric patients ≤17 years of age.

### Target audience

This guideline is intended for dietitians, nurses, pharmacists, physicians, advanced practice providers, and any other medical health professional involved in the nutrition care of infants and pediatric patients requiring a feeding gastrostomy tube.

## METHODS

### The panels

This guideline was composed of four panels: a clinical panel, a bias panel, a Delphi panel, and a validation panel. The clinical panel was chaired by Beth Lyman MSN, RN and comprised an international mix of ASPEN and non‐ASPEN members from the United States and Canada. The clinical panel included a balance of nurse practitioners, clinical nurse specialists, physicians, and surgeons. Specific areas of expertise included pediatric nutrition support nursing (BL, PG), pediatric surgery (LB, JK, DW), pediatric gastroenterology (RK), pediatric nurse practitioner practice spanning gastrointestinal, surgery, and advanced practice roles (KC, SO, EP, CM), pediatric emergency medicine and emergency ultrasound (CF), nutrition support clinical nurse specialists (PG, CM), and pediatric complex care medicine (GR). The role of the clinical panel was to create key questions using the Population, Intervention, Comparison, Outcome, and Timeframe (PICOT) framework, assist in the creation of search terms, perform the screening and data extraction, create the initial recommendations, and edit the final manuscript. The Delphi and validation panels were composed of the members of the clinical panel. They were split into two groups: one to perform the initial Delphi and one to validate the work of the initial group. The bias panel was composed of doctoral‐level researchers with a background in nutrition (SP, JM, DC). All panels were trained and overseen by an epidemiologist/methodologist (LM).

#### The protocol

An “a priori” protocol detailing the methods for this guideline and the key questions it would address was made available to the public for 2 months.^13^ Feedback was solicited from both the ASPEN database and externally through other relevant professional medical organizations. Comments were discussed by the clinical panel, and the protocol was adjusted where appropriate.

#### Study inclusion/exclusion criteria

To be included, an article had to be a study of pediatric patients less than or equal to 17 years of age, published after January 1, 2008, and have a primary or secondary objective that was relevant to at least one of the PICOT questions. The study design also had to permit meaningful causal inference. This resulted in a restriction to randomized control trials and quasi‐experimental designs, as well as validation studies where the patients served as their own controls. See the protocol for more information on design inclusion.^13^ All other studies were excluded from the formal analysis and decision making, but a separate discussion of select nonincluded studies was permitted when particularly relevant to the topic.

### The search strategy

**Figure 1:**
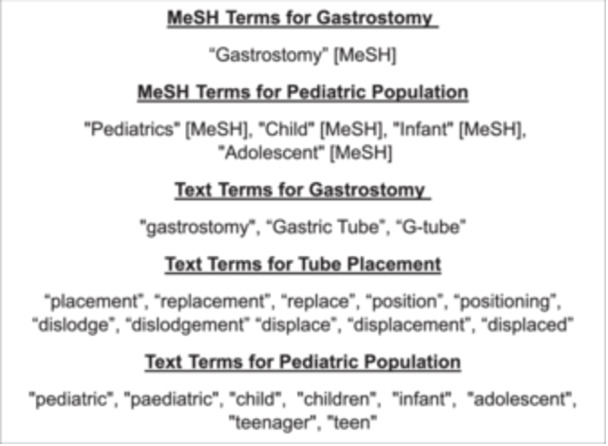
PubMED MEDLINE search strategy. MeSH, Medical Subject Headings.

The PubMED/MEDLINE, EMBASE, Cochrane Central, and CINAHL databases were searched from January 1, 2008 to October 5, 2025. We limited the search to 2008–present to reflect contemporary practice. Panel members noted that by roughly 2008 pediatric gastrostomy practice had moved toward more interventional radiology (IR)‐guided and laparoscopic placements, wider use of low‐profile tubes as the initial device, increasing overall gastrostomy utilization, and broader indications among medically complex children. The basic search strategy for PubMED/MEDLINE is given in Figure 1. Analogous strategies were conducted for EMBASE, Cochrane Central, and CINAHL.

### Data collection and presentation

The details for data collection are outlined in the published protocol and ASPEN Guidelines methods paper.^13^, ^14^ Briefly, all articles were screened and extracted in Covidence in duplicate by reviewers blinded to each other's decisions. Discrepancies were resolved by a third reviewer. The data were then converted into narrative tables.

### Bias analysis

The included studies were assessed for bias using the appropriate tools for their design. For quasi‐experimental designs, the Risk of Bias in Non‐randomized Study Interventions (ROBINS‐I)^15^ tool was used. For validation studies, the Quality Assessment of Diagnostic Accuracy Studies (QUADAS‐2)^16^ was used. Assessments were performed in duplicate by the members of the bias panel and discrepancies were addressed via consensus of the two bias assessors for that study.

### Statistical analysis

The plan for statistical analysis is detailed in our protocol.^13^ Due to the limited number of studies, no statistical analysis was conducted.

### Recommendations

**Figure 2:**
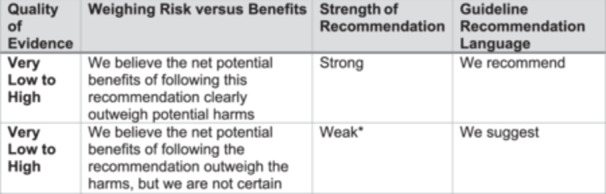
GRADE Language for Guideline Recommendations. *The designation of “Weak” is synonymous with the designation of “Conditional” that is currently being used in the new GRADE book. We have retained the term “Weak” because it more clearly represents the sentiment we intend to convey. GRADE, Grading of Recommendations, Assessment, Development, and Evaluation.

The Grading of Recommendations, Assessment, Development, and Evaluations (GRADE) was used to assess the quality of the available evidence and the strength of the recommendations (Figure 2).^17^ This process has been described in detail in other ASPEN guidelines.^18^ Briefly, GRADE provides a transparent road through the decision‐making process. The literature is assessed for its ability to answer the PICOT question. This produces a rating for Study Quality (Very Low/Low/Moderate/High). Based on those data and a qualitative weighing of the potential harms against the potential benefits of following the recommendation, a Strength rating is created (Weak/Strong). New terminologies have recently been introduced by the GRADE group, replacing the term “Weak” with the term “Conditional.” We have chosen not to adopt that alteration; however, the meaning of the two terms may be considered equivalent for the purpose of this guideline. To accomplish this, the clinical panel was convened multiple times to discuss, create, and refine the recommendations. Wherever inadequate data were available to guide a recommendation, the recommendation was labeled as Expert Opinion.

A modified Delphi approach with a validation panel was used to further refine the recommendations. The methods for this have been previously described.^14^ Briefly, this involved splitting the clinical panel into a Delphi panel and a Delphi validation panel. The Delphi panel took a blind vote on the recommendations and suggested edits. Edits were made as appropriate with a threshold agreement of 70%. The recommendations were then sent to the validation panel and the process was repeated.

## RESULTS

**Figure 3:**
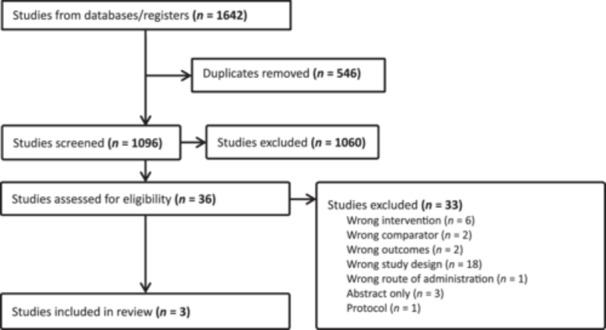
Prisma flow sheet.

The search strategy yielded 1642 Citations. Of these, 546 were duplicated and were removed. After review, 1639 were removed because they did not meet the inclusion criteria. This left three studies for data extraction and inclusion in this guideline (Figure 3). Due to the limited number of included studies, no statistical analysis was conducted.

### Routine replacement recommendations

#### Question 1

In infants and pediatric patients receiving routine replacement of a BGT, does confirming gastric tube placement via gastric aspiration with pH versus gastric aspiration without pH result in fewer adverse events?

*Recommendation*: In infants or pediatric patients with a BGT requiring routine replacement, we recommend that the gastric fluid be aspirated to confirm placement. Gastric pH measurement may not be necessary as a first‐line method.
*Certainty of evidence*: Very Low/Expert Opinion
*Strength*: Strong
*Delphi panel agreement*: 100%
*Validation panel agreement*: 100%
No studies met the inclusion criteria for this question.

##### Rationale and discussion

Clinicians and caregivers require a standard, accurate method for BGT replacement confirmation that is feasible and minimizes potential harms. There are several benefits to using gastric aspiration without pH as a first‐line method of BGT placement confirmation. It is simple, fast, cost‐effective, and may be performed using supplies already present in the replacement kit. This makes it ideal in both clinical and home settings. It avoids the added burden of creating point‐of‐care testing requirements, such as staff competency validation and pH strip storage. Its accuracy also reduces unnecessary escalation to radiologic confirmation. While no studies met our inclusion criteria, the accuracy of this method has been corroborated by one large, retrospective study (*n* = 1713) following pediatric patient BGT replacements over 18 years. This study confirmed that gastric aspiration without pH, while not highly specific (63.6%), did demonstrate high sensitivity (99.8%) for accurate replacement confirmation.^19^ The potential harms of this recommendation are that gastric aspiration without pH measurement could be less specific, and in rare cases may miss a case of misplacement of the BGT. However, these risks may be mitigated by close monitoring and escalation to pH testing or even radiologic imaging when misplacement is suspected. Overall, we feel that the benefits of this low‐burden, widely accessible method outweigh the potential harms.

Aspiration with pH measurement has also been reported to be accurate.^20^, ^21^ These two studies did not meet our inclusion criteria; one studied an adult population,^20^ and the other compared pH to other confirmation methods.^21^ Both reported the ability of pH measurement to correctly confirm BGT replacement. Measurement of the pH of gastric fluid is the current standard of practice for nasogastric feeding tube placement verification in pediatric patients in the United States and in all patients in the United Kingdom, but it is not without drawbacks and potential harms.^12^, ^22^ While we acknowledge that pH measurement can augment BGT replacement verification, we question the relative cost–benefit of this additional verification for several reasons. Measurement of gastric pH may be skewed if the child is receiving an acid‐suppressing medication or by the presence of enteral formula in the aspirated fluid.^21^ A more basic pH would require escalation to another confirmation method, which would likely involve radiation exposure. Additionally, pH measurement is considered point‐of‐care testing in many facilities, which requires annual validation of clinical competency for all staff, in addition to testing for color blindness. Storage of pH strips or paper requires protection from light and moisture. Access to supplies for checking pH levels may be problematic for caregivers at home. The current recommendation to make gastric aspiration without pH the first‐line method is based on the known efficacy of the technique and the potential harms, expense, and burden that could come from the sequelae of pH testing. To be clear, we are not recommending against pH testing. We are suggesting that it not be the first‐line method for tube confirmation.

When gastric aspiration without pH is inconclusive, other methods may be employed. Aspiration with pH is an option. Another simple option may be to aspirate gastric contents of the in situ BGT prior to BGT placement and compare appearances of pre‐replacement and post‐replacement gastric aspirates. Typically, gastric fluid contains mucus and has a greenish‐yellow tint, or what appears to be a diluted enteral formula. A retrospective study demonstrated ingestion of a colored beverage prior to BGT replacement as a potential cost‐effective method for confirmation via aspiration.^20^ Adult patients received a colored beverage instilled in the BGT in situ or consumed orally in 145 tube replacements. The colored beverage was visible in the aspirate or changed the color considerably in 95% of returns, making this a viable method for placement confirmation. Both of these latter techniques require forethought as fluid appearances are compared before and after BGT replacement.

##### Future research

We suggest future research be conducted to test the effectiveness of the supplemental measures suggested to confirm routine BGT replacement. Studies examining the use of a colored beverage instilled in the BGT prior to routine replacement would be important. Aspiration of fluid before and after routine BGT replacement to compare aspirate appearance should be examined as another method to confirm placement. Studies may also test comparisons of aspiration of gastric fluid obtained from a newly replaced BGT for pH measurement versus no pH measurement. Since many tubes are routinely replaced at home, a survey of families to discern how they confirm placement at home and how often they encounter complications such as inability to obtain a gastric aspirate would guide predischarge education efforts and would be useful. Outcome measurements for these studies should include BGT misplacement, use of radiologic contrast studies, and healthcare resource utilization.

#### Question 2

In infants and pediatric patients undergoing the initial replacement of a BGT, does waiting more time versus less time from initial placement result in fewer negative clinical outcomes?

*Recommendation*: In infants or pediatric patients with a BGT, we recommend waiting 6–12 weeks after BGT placement for the initial replacement.
*Certainty of the evidence*: Very Low/Expert Opinion
*Strength of recommendation*: Strong
*Delphi panel agreement*: 100%
*Validation panel agreement*: 100%
No studies met the inclusion criteria for this question.

##### Rationale and discussion

The current recommendation to wait 6–12 weeks after initial BGT placement for initial replacement carries the potential benefit of reduced complications, such as false tract formation or intraperitoneal placement.^11^ Waiting permits time for the tract to mature. While it is possible that longer wait times before initial replacement could lead to patients outgrowing stoma length, causing compression of the tract or pressure injury to the peristomal area, these risks can be mitigated through proper monitoring. Overall, we feel the benefits of minimizing the more serious complications of peritonitis and false tract formation outweigh the more manageable risks that come with waiting longer.

The question of timing for initial BGT replacement often depends on institutional policy and BGT placement techniques, such as the routine practice of gastropexy. Placement of a BGT creates a stoma between the layers of the epidermis, abdominal wall, and gastric mucosa. Over time, this heals to form a tract that is sealed and not easily penetrated. While there are no data in the literature documenting when a tract is “mature” or “established,” conventional practice suggests that a time period of 6–12 weeks after the BGT placement is adequate. This is thought to be sufficient time for most patients to then have the initial BGT replacement. The rationale for waiting a set amount of time before initial BGT replacement is to prevent inadvertent peritoneal BGT misplacement, also known as a false tract, which is more likely to occur just after BGT placement.

While there are no data to suggest that the timing of BGT replacement impacts adverse events, there are potential benefits and risks to both shorter and longer wait times. The benefit of waiting less time—that is, performing initial replacement on the lower end of the recommended 6–12 weeks—is that it provides the infant or child with less time to gain weight, possibly preventing a pressure injury. It also shortens the period during which caregivers must depend on professionals to replace the BGT. The risk of waiting less time is likely minimal unless the BGT procedure did not include suturing the stomach to the abdominal wall or the patient has risk factors for delayed healing. The benefit of waiting longer, meaning closer to 12 weeks, is that it better ensures that the tract is well established. The risk, however, is that it could result in compression of the tract from catch‐up weight gain and cause pressure injury if the BGT is low profile. If feasible, we suggest monitoring the patient for growth, pain, and discomfort in anticipatory fashion after BGT placement.

##### Future research

Research is needed to confirm the appropriate timing between BGT placement and initial replacement. A multicenter randomized controlled trial comparing patient outcomes after initial BGT replacement using two different timepoints, such as waiting 6 or 12 weeks, would inform practice. Relevant outcomes for this study should include tube misplacement, need for radiographic contrast studies, and stomal skin condition at the time of first replacement. It would also be of interest to compare patient outcomes with the use of gastropexy and the timing of the initial planned replacement of the BGT. Quasi‐experimental studies comparing patient outcomes using 6‐ or 12‐week time periods between different institutions would also be useful.

#### Question 3

In infants and pediatric patients receiving a routine replacement of a BGT and in whom gastric aspirate is not attainable, does confirming gastric tube placement via ultrasound (US) versus radiologic contrast study result in fewer negative clinical outcomes?

*Recommendation*: In infant or pediatric patients with a BGT, when gastric aspirate is not attainable, we suggest using US, when feasible, to verify gastric position post replacement. Failure to obtain a verification of correct BGT replacement using US requires a radiologic contrast study to confirm that the balloon is correctly placed within the stomach.
*Certainty of the evidence*: Very Low/Expert Opinion
*Strength*: Weak
*Delphi panel agreement*: 100%
*Validation panel agreement*: 100%
No studies met the inclusion criteria for this question in the original search.

##### Rationale and discussion

There are several potential benefits to using US to confirm BGT placement when gastric aspirate is unobtainable or when replacement is difficult. US avoids radiation exposure, can be performed rapidly in some settings, and may be less expensive. When performed by skilled clinicians, it has potential to be diagnostically accurate.^23^ However, the need for specially trained staff and access to equipment may limit feasibility. Despite this, we believe the benefits of providing radiation‐free confirmation for BGT replacement outweigh the potential need for increased staff or equipment burden. The potential harm from using US instead of a radiographic contrast study is the misinterpretation of the study due to human error, although this can also occur with a contrast study. Widespread acceptance of US will require the availability of trained staff to perform the procedure 24/7, which may be a limitation of its utility. The limitation is likely outweighed by the ability to confirm BGT replacement without radiation exposure and is the reason for our current recommendation.

The recommendation for use of US to confirm BGT placement when aspirate is unavailable is contingent on how the term “feasibility” is defined and whether other measures have failed, such as rolling the child on their side (usually left) and waiting a few minutes to obtain gastric aspirate. For the purpose of using US to confirm replacement BGT position, “feasible” means that the institution has trained staff with proficiency in using US in this manner. Each institution will need to define the parameters for training staff in this procedure.

One small validation study in BGT replacement following tube dislodgement provides an opportunity to extrapolate findings to our population. Fifty children who presented to the ED with a dislodged BGT served as study subjects.^23^ In this study, after BGT replacement, Pedialyte or normal saline was instilled into the new BGT during the US procedure to ensure visual confirmation of the fluid draining into the stomach as opposed to the peritoneum. This study compared US to radiologic contrast study results and found 96% sensitivity and 100% specificity in the 48 subjects who had both US and radiologic contrast study.^23^ There was one misplaced BGT and it was identified by US in this study.

A prospective, quasi‐experimental study compared the accuracy of Point‐of‐Care Ultrasound (POCUS) and radiologic contrast study for gastric confirmation of routine BGT and the gastric balloon of the gastrojejunal (GJ) tube replacement. Of note, the investigators also evaluated results of US for novice and more experienced POCUS operators.^21^ This study looked at 118 (*n* = 104 routine replacements) GJ or BGT replacements that were a convenience sample recruited in IR and found that 13.6% were deemed indeterminate, meaning visualization of the gastric balloon was difficult or impossible.^21^ Of those 16 GJ/BGT indeterminate results, four tubes were confirmed by radiologic contrast study to be displaced into the stomal tract. Comparing POCUS with radiologic contrast study found fair agreement between POCUS and radiologic contrast study, depending on the expertise of the individual performing the POCUS, with less experienced POCUS operators reporting more indeterminate placements, with 87% sensitivity for POCUS and no specificity reported due to the low number of misplaced tubes.^21^ While not one of the primary specific aims of this study, investigators reported that 88 of the 118 tube replacements yielded a gastric aspirate with a pH measurement of 4.1.^21^ This study validates our premise that staff training and proficiency may well impact the utility of POCUS in this clinical scenario.

##### Future research

More research is needed to validate the agreement between US and radiologic contrast for confirming routine tube replacement when an aspirate cannot be obtained. This would require a much larger sample size and independent replication of the high sensitivity. Further, this should be performed in patients receiving a routine BGT replacement. A well‐designed diagnostic accuracy study comparing US to radiologic contrast study would add important evidence for this emerging novel practice. Performing routine BGT replacements in a clinic using a point‐of‐care approach versus having the replacement done in the radiology department, with a comparison of patient outcomes and costs, would help clinicians determine the utility of this technique when unable to obtain a gastric aspirate from a newly replaced BGT.

#### Questions 4 and 5 combined

Question 4: In the infant and pediatric patient receiving routine replacement of a BGT who also has a peritoneal dialysis (PD) catheter or ventriculoperitoneal (VP) shunt, does confirming gastric tube placement via aspiration (with or without pH) versus radiologic contrast study result in fewer negative clinical outcomes?

Question 5: In the infant and pediatric patients receiving routine replacement of a BGT who also has a PD catheter or VP shunt, does waiting more time versus less time post initial tube placement result in fewer negative clinical outcomes?

*Recommendation*: In infants or pediatric patients with a VP shunt requiring routine replacement of a BGT, we recommend aspiration of gastric fluid as the first‐line method to confirm gastric placement.
In infants or pediatric patients with a PD catheter requiring routine replacement of a BGT, where there is no fluid leakage onto the abdomen from around the stoma, we recommend aspiration of gastric fluid as the first‐line method to confirm gastric placement.
In infants or pediatric patients with a BGT and PD catheter or VP shunt receiving routine initial replacement of the BGT, we recommend waiting a longer time—that is, closer to the previously mentioned 12 weeks post placement—if peristomal fluid is leaking onto the abdomen. If feasible, waiting until the peristomal area is without fluid leakage is ideal. Otherwise, in the event of fluid leakage, we recommend following the institutional policy for when initial routine BGT replacement is performed.
If replacement is indicated or necessary, we recommend observing the appearance of gastric aspirate before and after BGT replacement to compare for similarity of appearance. Alternatively, we recommend instilling a small amount of a colored beverage into the existing BGT prior to removal to compare fluid aspirated from the newly placed BGT for similarity of appearance. If indicated, or previous methods have been inconclusive, we recommend obtaining a radiologic contrast study to confirm placement of the newly replaced BGT. For institutions with proficiency in using US for BGT replacement verification, this would be preferred over a radiologic contrast study.
*Certainty of evidence*: Very Low/Expert Opinion
*Strength of recommendation*: Strong
*Delphi panel agreement*: 100%
*Validation panel agreement*: 100%
No studies met the inclusion criteria for this question.

##### Rationale and discussion

These two questions are combined as they have similar concerns regarding both initial replacement verification and timing of the initial replacement, due to the unknown impact of either peritoneal dialysis fluid or cerebrospinal fluid on maturation of the BGT tract. Both clinical situations can potentially result in increased abdominal distension, which could theoretically delay BGT tract maturation. While the benefit and risk assessments may differ slightly, both focus on the intent to prevent or accurately identify when a false tract may occur with initial routine BGT replacement. Though not initially considered in our questions, ascites could be considered similarly, as it is also peritoneal fluid, which could affect tract maturation.

In children with a VP shunt or PD catheter, using gastric aspiration for BGT placement confirmation, rather than radiologic imaging, offers the benefits of reduced radiation exposure and reduced cost. The ability to quickly verify placement of a replaced BGT shortens time in the healthcare setting for the family, quickly assures placement, and rules out complications such as misplacement. The potential harm is that a misplaced tube might still yield fluid, albeit from the peritoneum, which would be incorrectly verified as gastric placement. This may be mitigated through escalation to radiologic imaging if peristomal leakage is observed, the findings of aspiration are inconclusive, or other concerns arise. For this reason, we recommend a stepwise escalation approach balanced with clinical judgment that begins with gastric aspiration as the first‐line method.

The benefit of waiting more time versus less time is contingent upon whether or not peristomal leakage is seen. Waiting more time—that is, until no fluid leakage is apparent—suggests that the BGT tract is sufficiently mature to allow for initial BGT replacement. Periostomal leakage during the initial BGT may suggest that the tract is not yet mature. This increases the risk that blind replacement might cause a false tract. This could lead to aspiration of fluid that might look like gastric contents when, in fact, it is not. A subset of patients not previously addressed are those with a newly placed BGT and ascites where peristomal leakage is also an ongoing concern. An additional measure to confirm gastric placement would be to place a colored beverage in the in situ BGT to compare the color of aspirated fluid from the newly replaced BGT.

We suggest that the best way to confirm placement of a BGT in children with extenuating circumstances, such as presence of a PD catheter or VP shunt, is to balance the risk of delayed or disrupted tract integrity and possible false tract formation with the benefit of using a less invasive method to confirm placement. Any clinical sign of tract integrity disruption, such as fluid or blood seeping around the stoma, should escalate the method(s) used to verify BGT placement. The current recommendations reflect the need for the clinician to pause prior to routine BGT replacement and consider whether the timing is optimal and if further maneuvers should be used to confirm placement beyond obtaining a gastric aspirate. We acknowledge that a radiologic contrast study should be performed if deemed necessary by the clinician's risk assessment.

While no data met our inclusion criteria, three studies may shed some light on the current recommendation, the findings of which are consistent with our rationale. It is common practice for many children who require a VP shunt or PD catheter to also have a BGT.^24^, ^25^, ^26^ Three retrospective studies examined infection risk when a child had both a BGT and a VP shunt or PD catheter. While one reported a correlation between peritonitis and BGT placement with PD catheter use,^26^ two found no correlation between infectious complications and BGT placement with a VP shunt.^24^, ^25^ Importantly, none of these studies reported complications related to BGT replacement or confirmation. These medical devices, required to treat kidney or neurologic conditions, involve fluid in the abdomen that could affect girth and BGT tract maturation.

##### Future research

Well‐designed diagnostic accuracy studies are needed comparing gastric aspiration with other methods of placement confirmation in patients with either a VP shunt or PD catheter. Gastric aspiration should be compared with pH measurement or use of a colored beverage that is instilled or consumed prior to replacement. Randomized control or quasi‐experimental trials to examine the efficacy of shorter versus longer wait times after initial BGT placement for initial BGT replacement in infants and children with a VP shunt or PD catheter would inform practice. Quasi‐experimental trials of supplementary methods to verify BGT replacement in this population would also be useful.

#### Question 6

In infants or pediatric patients receiving routine initial replacement of a BGT, where the patient has conditions that could adversely affect stoma maturation such as kidney disease requiring a PD catheter, neurologic condition requiring a VP shunt, an oncologic condition requiring chemotherapy, diabetes, or a history of chronic steroid use, does waiting more time versus less time post initial placement result in fewer negative clinical outcomes?

*Recommendation*: In infants and pediatric patients who have both a BGT that requires initial replacement and an extenuating comorbidity that may delay wound healing and, therefore, tract maturation, we suggest waiting longer—that is, closer to 12 weeks post placement—to perform the initial BGT replacement. We also suggest not changing the BGT if there is peristomal drainage such as fluid or blood, if possible.
*Certainty of evidence*: Very Low/Expert Opinion
*Strength*: Weak
*Delphi panel agreement*: 100%
*Validation panel agreement*: 100%
No studies met inclusion criteria for this question.

##### Rationale and discussion

The benefit of waiting longer to perform initial BGT replacement in the above‐mentioned patient scenarios is to allow more time for tract maturation when the impact of these comorbidities on tract healing is unknown. If it is possible to wait longer than 6–8 weeks post initial BGT placement for BGT replacement, the tract may be healed enough to allow for this procedure to be done without complication. The primary complication and risk factor for initial BGT replacement in this patient population is false tract formation during the replacement procedure. The patient population most at risk for this to occur would be a child who tenses their abdominal muscles during the procedure, requiring the clinician to apply increased pressure to replace the BGT. It is challenging to predict which children will cooperate during the initial BGT replacement, so preemptive mitigation measures, such as distraction, should be employed.

While there is some evidence in animal models that tract maturation occurs as early as 1 week, this does not account for comorbidity status, and there are no data to corroborate this in humans.^27^ Delayed tract maturation is a risk of BGT replacement in this population. Children with these comorbid states may benefit from observation for pain or abdominal distension with the first use of the newly placed BGT.

##### Future research

High‐quality human studies evaluating the impact of comorbidities on tract maturation following initial BGT replacement are urgently needed. RCT's or quasi experimental trials should assess the optimal timing for BGT replacement in infants and pediatric patients with conditions known to impair wound healing. This research should aim to determine whether extended time to replacement improves clinical outcomes and reduces complications, such as leakage, tube dislodgement, and tract disruption.

#### Question 7

In infants and pediatric patients receiving a routine replacement of the initial BGT, does the use of a care bundle compared to the nonuse of a care bundle result in fewer negative clinical outcomes?

*Recommendation*: In infants and pediatric patients with a routine BGT replacement, we recommend the use of a care bundle to standardize and manage patient care, including caregiver education post BGT replacement.
While we acknowledge that each institution will customize specific information provided to caregivers and older children with a newly placed BGT, we recommend that the elements of the care bundle mimic those in the literature. The components that should be included in a care bundle for caregiver education (including the older child with a newly placed BGT) include: who is permitted to perform initial and subsequent routine BGT replacement; the time period between placement and replacement; what education is provided to caregivers; how that education is provided; and how healthcare literacy is addressed. Other components of the care bundle that deal with patient assessment before BGT placement should include: delineation of the process for initial BGT replacement, including pre‐procedure evaluation and determination of the preferred BGT placement method and setting; an algorithm for evaluating patients presenting to a healthcare facility for nonroutine BGT replacement; and complication management with information on whom to call for help when their regular staff resources are not available. These elements provide a comprehensive care bundle that clinicians can use to standardize care of the child who requires a BGT replacement.
*Certainty of evidence*: Very Low/Expert Opinion
*Strength of recommendation*: Strong
*Delphi panel agreement*: 100%
*Validation panel agreement*: 100%
No studies met our inclusion criteria for this question.

##### Rationale and discussion

While care bundles are not a new concept to healthcare, the term warrants some definition as it pertains to BGT replacement care. A care bundle is defined as a small set of interventions developed by a multidisciplinary team to improve care provided to a defined patient population.^28^ This concept is based on using evidence‐based practices to decrease variability in the care of the patient population with the intent to improve patient outcomes. Care bundles can be national efforts or institution based, but all rely on adherence for success.^28^

The current recommendation is based on the obvious benefit of patient and caregiver education. Caregivers and patients need tools that optimize their knowledge on how to deliver nutrition safely and respond to common problems. Further, there are no significant known harms with the provision of a care bundle. There are several studies that, while they did not meet our inclusion criteria, corroborate our viewpoint on the benefit of providing a care bundle.^29^, ^30^, ^31^, ^32^, ^33^, ^34^ The following studies did not meet our inclusion criteria for this question because none of them specifically addresses routine BGT replacement.

The components for inclusion in the above recommended care bundle are based on results from studies showing that pre‐BGT placement evaluation, standardized nursing care, and standardized caregiver education improve patient outcomes. Two pre‐care and post‐care bundle implementation studies document improved patient outcomes such as decreased length of stay (LOS) and ED visits.^29^, ^30^ Both of these studies addressed pre‐BGT placement patient assessment, nursing care, and post‐discharge needs, but not routine replacement as was part of our search criteria. One study developed a Gastrostomy Readiness checklist while the other study defined the pre‐procedure patient evaluation. Two studies comparing patient outcomes before and after implementation of a BGT caregiver education program reported significant decreases in utilization of healthcare services, including office visits and ED visits.^31^, ^32^ The benefits of caregiver education post BGT placement were sustained 1 year post discharge, as demonstrated by decreased office and ED visits.^32^ The benefit of these care bundles for caregivers was detailed in two studies that described decreased anxiety, improved quality of life, and better understanding of BGT management post initiation of a standardized discharge education program post BGT placement.^33^, ^34^

Three other studies contribute useful context for this question. A large report using Pediatric Health Information System data examined unplanned ED and hospital visits among the 30 most common pediatric surgical procedures.^35^ This study identified BGT placement as having double the number of ED visits and significantly higher associated costs and LOS.^35^ The authors suggest efforts be focused on post‐procedure care and discharge education.^35^ One qualitative study of “high‐performing” pediatric institutions that place BGTs looked for themes that inform BGT post discharge patient success.^7^ These themes included education on what to expect, availability by phone call or clinic visit to address issues and avoid the ED, and empowerment of caregivers to manage routine BGT‐related problems.^7^ High performing institutions had low 30‐ or 90‐day readmissions or ED visits. Another aspect of caregiver education is where caregivers look for information, which may include the internet. A recent publication evaluated 229 BGT‐related videos on YouTube and found that just over half (50.7%) were considered high quality.^36^ Half of the videos from independent content creators, as opposed to nurses or hospitals, were considered low quality.^36^ The authors recommend clinicians help guide caregivers to vetted internet resources.

Our recommendation is based on the demonstrated benefits of using a care bundle, without documented evidence of harm. We believe any process that standardizes care, management, and caregiver education around BGT replacement is beneficial to families and uses fewer healthcare resources. Once a care bundle is implemented, periodic monitoring of adherence and patient outcomes is warranted.

##### Future research

We acknowledge that there is sufficient evidence to support care bundles for initial BGT placement. However, there are currently no studies on the use of care bundles for routine BGT replacement. Descriptive and quasi‐experimental studies on patient outcomes related to a care bundle for routine BGT replacement would help further inform practice for this particular question. These studies should look at the cost savings of decreased utilization of healthcare resources to validate the cost effectiveness of care bundles.

#### Question 8

In infants and pediatric patients receiving a routine replacement of a BGT, does the use of formal focused clinician education versus no formal focused clinician education result in fewer negative clinical outcomes?

*Recommendation*: In infants or pediatric patients with a BGT, we recommend implementation of a staff education program focusing on routine replacement with didactic and supervised components.
We recommend that the elements of the program should include, at a minimum, patient assessment, BGT stoma site and size assessment, routine replacement, routine verification of the replaced BGT, signs and symptoms for escalation to elicit support from more senior staff or more invasive interventions, replacement evaluation using nonroutine methods, and protection of the BGT from accidental or premature dislodgement. Supervision of staff who are being trained on routine BGT replacement is recommended, but can be institution specific in terms of the number of supervised routine BGT replacements.
*Certainty of evidence*: Very Low/Expert Opinion
*Strength of recommendation*: Strong
*Delphi panel agreement*: 100%
*Validation panel agreement*: 100%
No studies met our inclusion criteria for this question.

##### Rationale and discussion

The primary benefit of this recommendation is that a well‐educated staff will be more competent and consistent in care. This should lead to fewer errors, safer procedures, and reduced emergency visits. It also instills confidence in the caregivers. An educated staff will be better equipped to recognize when escalation is necessary, which should prevent complications. The harms of such a program, such as loss of time for education, are minimal and certainly outweighed by the benefits. Our recommendation follows the healthcare trend toward competency validation for common but potentially complex procedures.

A recent study that did not meet our inclusion criteria, as it focused on dislodged BGTs, compared the average LOS in a pediatric ED for children requiring BGT replacement that was performed by nurses who were specially trained versus physicians.^37^ The average LOS for the 58 children with BGT replacement by a nurse was 22 min, compared with 86 min for the 52 subjects who had a physician perform BGT replacement (*P* < 0.0001).^37^ LOS may not be considered a negative clinical outcome by clinicians, but shorter LOS in an ED is less disruptive to the child and family. This study demonstrates the benefit of expanding the role of nurses, post special training, for dislodged BGT replacement.

Other studies failing to meet our inclusion criteria for this question, as they do not address routine BGT replacement, similarly offer context to validate the benefits of clinician education. Two pediatric institutions noted inconsistent content being provided to caregivers post BGT placement and developed multifaceted clinician education programs that included education sessions, education resource materials, and the use of unit‐based champions to facilitate implementation.^34^, ^38^ Both of these studies focused on a formal nurse education program for caregiver BGT instruction and demonstrated improved knowledge among caregivers. One of these studies documented a decrease in ED visits but not unplanned clinic visits post implementation.^38^ Another study reported the benefits of nurse education in an inpatient care bundle to minimize post‐BGT complications.^29^ Nursing staff education efforts focused on prevention of accidental access of the BGT port, protection of the accessed BGT from pulls or tugs, and disconnection of the low‐profile BGT extension set when not being used.^29^ Investigators documented a reduction in inpatient BGT dislodgement from 14% to 1.5% post nursing bundle implementation. The authors acknowledge their inability to attribute their improved outcomes to a single intervention but attribute success to the interdisciplinary approach to this quality improvement project and ongoing process evaluation.

The recommendation for staff education is based on these potential benefits and no evidence of harm. Replacement of a BGT may seem routine, but there are many nuances where a clinician should use critical thinking skills. Education programs can help staff attain these skills. Didactic education is insufficient to teach the skills associated with routine replacement of a BGT; supervised BGT changes are also necessary. When teaching a clinician how to replace a BGT, a checklist of “steps” may be helpful to evaluate competency, with a requirement of a certain number of replacements in the presence of a mentor before being permitted to do it independently. Another consideration is to determine “who” can perform a routine BGT replacement. Is it within their scope of practice? Does the organization in which they work support/allow them to change tubes? We suggest organizations have a written plan and implementation process for staff education to perform routine BGT replacement, with the required number of annual BGT replacements to maintain competency. While there are no data to support what number of BGT replacements are needed to maintain ongoing competency, regularly practicing this skill does increase confidence and comfort in performing this procedure safely and independently.

##### Future research

Future studies should evaluate the effectiveness of formal clinician education. While randomizing one group to no clinician education would not be ethical, other observational and quasi‐experimental designs might be pursued. This could include stepped‐wedge studies, quasi‐experimental cluster‐controlled trials, or a quasi‐experimental retrospective design examining our population at a hospital pre‐policy and post‐policy change. Research that considers which components of education are most effective should also be considered. Relevant outcomes for these studies include BGT dislodgement rates, infection, complications related to misplacement, and staff confidence and competence in BGT replacement. Research on how this clinician education is done—that is, online versus in‐person classes or using a simulation lab—would guide institutions to efficiently and effectively educate staff.

### Guidelines for nonroutine replacement

#### Question 9

In infants and pediatric patients with a BGT that inadvertently comes out before the tract is considered established, does confirming placement of the gastric replacement tube via a radiologic contrast study versus aspiration of gastric contents with or without pH result in fewer negative outcomes?

*Recommendation*: In infants and pediatric patients with a BGT that becomes dislodged before the tract is considered mature or established, we recommend confirming the placement of the new BGT via a radiologic contrast study. Institutions with capability and proficiency in US might utilize this technology instead of a radiologic contrast study as the first‐line method for confirmation of the newly replaced BGT in this clinical situation, when feasible.
*Certainty of evidence*: Very Low/Expert Opinion
*Strength of recommendation*: Strong
*Delphi panel agreement*: 100%
*Validation panel agreement*: 100%
No studies met our inclusion criteria for this question.

##### Rationale and discussion

This question balances the risks of radiation exposure and longer LOS against the benefit of definitively knowing that a high‐risk replaced BGT is in the correct position. Radiologic contrast provides a high degree of accuracy when confirming BGT replacement. This is the major benefit of this recommendation. It avoids potential serious consequences, such as unrecognized false tract placement and intraperitoneal feeding. Patients with immature tracts are especially vulnerable to misplacement because healing with scarring to “seal” the passageway from the abdomen to the stomach for the BGT may not be complete. Patient‐specific parameters, such as when the BGT was initially placed, how long it was displaced before replacement, and patient cooperation, may increase the risk of BGT misplacement further. The known harm of contrast studies is exposure to radiation, which has a cumulative effect. For this reason, despite the radiation exposure that accompanies radiologic contrast studies, we feel the benefits outweigh the harms and support the current recommendation.

Three reports in the literature did not meet our inclusion criteria but corroborate our rationale and may be worthy of extrapolation to our population. A report from the Pennsylvania Patient Safety Authority describes dislodged or possibly dislodged BGT in 183 patients aged 0–20 years old.^39^ Of the 1026 total dislodged BGTs (not stratified by patient age), nine deaths, two near deaths, and five patients with unrecognized false tracts were documented. This report lends credence to the concern that a real complication of inadvertent BGT removal and replacement may result in unrecognized false tract formation with the tip of the BGT in the peritoneum. Two retrospective studies reported BGT misplacement and complications from BGT replacement, along with the use of radiologic contrast studies.^6^, ^40^ Of the 237 subjects in the first study, 17 had a BGT replacement <60 days post placement and, of those, 15 had a radiologic contrast study with no false tracts observed.^6^ In a study of 337 children who presented to the ED for BGT replacement, subjects were categorized as symptomatic or asymptomatic and whether the BGT was in place for <45 days or >45 days.^40^ Two of the subjects who required BGT replacement at <45 days were found by radiologic contrast study to have false tract placement of the new BGT. Both studies recommended a radiologic contrast study be done if the BGT was in place <60 or <45 days, respectively. The authors developed an algorithm that recommends a radiologic contrast study be performed on all patients presenting to the ED for BGT replacement where: no fluid can be aspirated and the tube has been in place >45 days; BGT is <45 days old; or the patient has one or more of the above‐listed symptoms. These studies validate the utility of radiologic contrast studies for subsets of children with a BGT, especially when the tract is not considered mature.

##### Future research

Studies are needed to determine the safest and most effective strategies for confirming the placement of BGTs that become dislodged prior to tract maturation. Comparative studies that confirm placement via radiologic contrast versus simpler bedside techniques could inform best practices, but randomization may not be ethical if it leaves one group less certain of safe placement. Given this concern, quasi‐experimental retrospective designs using registry data to compare patient outcomes pre‐policy and post‐policy change or comparisons between hospitals with different policies may be a better starting point. Ideally, the number of days post BGT initial placement would be consistent as a secondary measure when doing these studies.

#### Question 10

In infants and pediatric patients with a BGT dislodgement (traumatic or accidental) after tract maturation, does confirming placement of the gastric replacement tube via aspiration of gastric contents with or without pH versus a radiologic contrast study result in fewer negative clinical outcomes?

*Recommendation*: In infants or pediatric patients with a mature tract where the BGT becomes dislodged accidentally (i.e., tube falls out with balloon deflated) or traumatically (i.e., tube removed with balloon inflated), we recommend aspiration of gastric contents over the use of a radiologic contrast study as a first‐line method of BGT confirmation.
*Certainty of evidence*: Very Low/Expert Opinion
*Strength of the recommendation*: Strong
*Delphi panel agreement*: 100%
*Validation panel agreement*: 100%
No studies met our inclusion criteria for this question.

##### Rationale and discussion

Gastric aspiration offers the benefits of being quick, cost effective, noninvasive, and highly practical for replacing a dislodged BGT. When performed by experienced clinicians or caregivers, it is typically sufficient to verify BGT placement. The potential harm is the risk of misinterpretation of a misplaced BGT, but we consider this to be rare in this population. It can be mitigated through a careful assessment of the patient history, cause of dislodgement, and timely monitoring of symptoms, particularly during the first use of the newly replaced BGT. When concern arises, escalation to radiologic imaging should be done. Given the simplicity and safety of gastric aspiration in the patient with a mature tract, we feel that the benefits of our suggestion to make aspiration of gastric fluid the first‐line treatment outweigh the potential harms.

Accidental or traumatic BGT dislodgement is common in children with a BGT and occurs at home, school, and healthcare facilities alike, but there is a paucity of literature to guide practice regarding how to confirm placement of the newly replaced BGT. One retrospective study that did not meet our inclusion criteria looked at the use of radiologic contrast studies done in 76 of the 337 subjects who required BGT replacement for dislodgement of a tube in place for >45 days and found that 4% were misplaced in asymptomatic children.^40^ Despite these data, we believe aspiration of gastric contents should be the first method used to confirm placement in this clinical scenario, based on our benefits versus risk of harm analysis.

The management and replacement confirmation for the newly replaced BGT should take into consideration the skill set of who is evaluating the situation, as well as the cause of the dislodgement. For example, a traumatically dislodged BGT should prompt concern about possible tract disruption due to trauma from the inflated balloon. We acknowledge that there are situations when a radiologic contrast study is the safest method for confirming placement. However, the majority of BGT replacements, in the context of accidental or traumatic displacement, can have accurate gastric position confirmation of the newly replaced tube using aspiration of gastric fluid. Clinician assessment of the BGT, along with patient history, will assist decision making regarding whether the use of standard of care to confirm placement of the replaced BGT or escalation to another method is needed. One subgroup not listed in the literature are children who frequently remove their BGT for self‐stimulation or attention. The result of frequent traumatic BGT removals could be disruption of the integrity of the tract, which could allow the BGT tip to end up in the peritoneum. This subgroup might warrant escalation of confirmation method beyond aspiration of gastric fluid. Finally, if feasible, we suggest monitoring the infant or child with a BGT replacement post accidental or traumatic dislodgement for pain when the new BGT is first used for feeding.

##### Future research

This clinical scenario is apt to happen multiple times to any child who has a BGT. Therefore, research involving caregivers as data collectors would be beneficial. Other important research questions include how often tube dislodgement happens outside a healthcare facility, what predisposing factors led to the dislodgement, how the placement of the replaced BGT was confirmed, and whether adverse events occurred, such as false tract or inability to replace the BGT at home. A large, multicenter study comparing results in patients with traumatic dislodgement who undergo a contrast study versus gastric aspiration would inform practice. Also of use would be quasi‐experimental designs examining before and after implementation of algorithms delineating who should and should not get a radiologic contrast study in this clinical situation.

#### Question 11

In infants and pediatric patients with a BGT that is difficult to replace, does confirming placement of the gastric replacement tube via a radiologic contrast study versus aspiration of gastric contents with or without pH result in fewer negative outcomes?

*Recommendation*: In infants or pediatric patients with a mature tract and BGT that was difficult to replace but did not require dilation, we recommend aspiration of gastric fluid with or without pH as the first‐line method to confirm placement of the replaced BGT. If gastric aspirate is unable to be obtained, it may be appropriate to use a radiologic contrast study to confirm placement of the newly placed BGT. Where US of the BGT is an established practice and feasible, it could be used to verify the newly placed BGT instead of a radiologic contrast study, thereby avoiding radiation exposure.
*Certainty of evidence*: Very Low/Expert Opinion
*Strength of recommendation*: Strong
*Delphi panel agreement*: 100%
*Validation panel agreement*: 100%
No studies met our inclusion criteria for this question.

##### Rationale and discussion

For the purposes of this guideline, a difficult‐to‐replace BGT is defined as one that cannot be completed smoothly with standard techniques and requires repeated attempts, increased resistance, or additional clinical concern, but that does not require tract dilation or operative assistance. While no data were found to answer this question, the current recommendation is based on the idea that difficult BGT replacement does not necessarily mean that harm has been done to the stomal tract, which could increase the risk of misplacement. In healthcare settings, homes, schools, or other environments, a consistent approach to confirming placement of the replaced BGT is beneficial after a difficult replacement. Thus, we suggest aspiration of gastric contents be used first, as it is the current standard of practice, and, if unsuccessful, escalate to US or a radiologic contrast study. The benefit of tube site confirmation that a radiologic contrast study offers outweighs the risks of radiation exposure to the patient when gastric aspirate is not obtained. The one exception might be to favor the use of US, if feasible, thereby avoiding radiation exposure. We acknowledge that a radiologic contrast study is the “gold standard” for definitively identifying BGT placement, and for patient safety this is the best approach when replacement is difficult and no gastric fluid can be aspirated, or US results are inconclusive.

In this clinical scenario of confirming placement of a difficult‐to‐replace BGT, it may seem safer to obtain a radiologic contrast study, but reports in the literature validate that this is a common occurrence for children presenting to the ED, and there is growing interest in protecting children from unnecessary radiation exposure. Two studies did not meet our inclusion criteria but warrant mention. A retrospective study looking at reasons radiologic contrast studies were performed in children with a BGT presenting to the ED for replacement identified a statistically significant difference (*P* < 0.001) in children who had difficult versus not difficult replacement and the use of a radiologic contrast study, which supports our recommendation.^6^ The investigators noted that children who had a radiologic contrast study also had a significantly longer ED LOS without a clear benefit from this escalated procedure, as opposed to using gastric aspirate. A prospective study describing the use of US compared to radiologic contrast study to verify BGT placement documented that 25 of the 50 subjects had difficult replacements.^23^ Both of these studies tout the benefits of using methods without ionizing radiation to confirm the placement of a replaced BGT.

The current PICOT question raises an important issue of how to define the term “difficult replacement.” No study we reviewed defined this term, and there is likely to be wide variation in this definition among clinicians. One study documented that surgeons are more likely to order a radiologic contrast study in the ED than the ED staff.^41^ However, that outcome was after implementation of an algorithm suggesting a surgery consult for more high‐risk, difficult replacements, which often result in escalation of confirmation method. Most clinicians with expertise in BGT replacement and management rely on their experience and critical thinking skills to guide decision making on this issue on a case‐by‐case basis. The term “difficult replacement” needs to be better defined using parameters such as patient pain, need for tract dilation, length of time to replace BGT, appearance of blood and injury at the stoma, and inability to obtain gastric aspirate after placement.

A second issue that warrants discussion is the lack of documentation of many aspects of BGT replacement in a patient's medical record, including the difficulty of the tube replacement. Two retrospective studies looking at children with a dislodged BGT that presented to the ED for replacement reported poor documentation, including how the newly replaced BGT was confirmed to be in the stomach.^6^, ^42^ This prompted one of these institutions to develop a template for documentation of BGT replacement. Of note, there is no section on difficulty of replacement. We suggest that any template used to document BGT replacement should include a place to record the subjective difficulty of the procedure. We also suggest that the patient be monitored during the first use of the new BGT for feeding tolerance prior to discharge from the ED.

##### Future research

Large quasi‐experimental studies that compare US and radiologic contrast studies with gastric aspiration to confirm difficult placements of newly replaced BGT are needed. Descriptive studies reporting on the frequency of difficult insertions that do not yield gastric aspirate are also needed. Finally, to facilitate better study, it would be helpful, as a field, to agree on a common definition for the term “difficult insertion.” Also, large multicenter studies in children with difficult BGT replacements that use the same decision algorithm to guide confirmation method decisions would better inform practice. Outcomes of these studies should also include economic outcomes such as cost of care and ED LOS. An additional question might be: Does offering a colored beverage in those who are safely able to drink, as compared with those who cannot drink colored beverage, improve verification of a newly replaced BGT?

#### Question 12

In infants and pediatric patients with a BGT that is difficult to replace and requires the use of a dilator, does confirming placement of the gastric replacement tube via radiologic contrast study versus aspiration of gastric contents result in fewer negative outcomes?

*Recommendation*: In infants and pediatric patients with a BGT and mature tract that required dilation prior to replacement, we suggest first attempting gastric aspirate and, if there is any concern about placement, obtaining a radiologic contrast study to confirm placement of the newly replaced BGT.
Where US of the BGT is an established practice and feasible, it could be used to verify the newly placed BGT instead of a radiologic contrast study, thereby avoiding radiation exposure.
*Certainty of evidence*: Very Low/Expert Opinion
*Strength of the recommendation*: Weak
*Delphi panel agreement*: 100%
*Validation panel agreement*: 100%
No studies met our inclusion criteria for this question.

##### Rationale and discussion

While no data were identified to directly answer this question, our recommendation is based on the benefit of knowing definitively where the replacement BGT is located anatomically outweighing the risks associated with radiation exposure, increased ED LOS, and cost. Dilation of the stomal tract can disrupt its integrity and allow for false tract formation by the replacement BGT. This may not be evident clinically because the dilation procedure is painful even when no harm is done to the tract. Dilation is a frequent procedure required for BGT replacement in the ED, and disruption of the tract may not be apparent. We, therefore, suggest that a radiologic confirmatory study be obtained *if* unable to first obtain gastric aspirate.

As with the previous question, stomal dilation may lead a clinician to obtain a radiologic contrast study routinely, though this approach has been disputed in the literature. Three retrospective studies that did not meet our inclusion criteria document the frequency of BGT stomal dilation to be between 32% and 53% of all children who came to the ED for BGT replacement.^6^, ^42^, ^43^ One study reported methods used to confirm placement for the replaced BGT, including aspiration of gastric contents (58%) and radiologic contrast study (40%), along with evaluation of feeding tolerance (34%), auscultation of air (18%), and abdominal examination (2%).^43^ Of 97 dilation procedures, one replacement BGT was found to be misplaced. These investigators did not recommend escalation of tube placement confirmation method in the setting of serial dilation of a stenosed, mature stomal tract. Another study documented that 46% of the children who required BGT stomal dilation had a radiologic contrast study done post replacement. None of these studies recommended routine use of radiologic contrast studies as the first method to confirm placement of a replaced BGT post stomal dilation.^6^

##### Future research

Further research is needed to evaluate the safety and accuracy of BGT placement confirmation methods after tract dilation. Comparative effectiveness studies that assess radiologic contrast, gastric aspirate, and US would help define the approach that is most reliable and least invasive. Relevant outcomes include misplacement rates, complications, time to feeding, cost of the interventions, and exposure to radiation.

#### Question 13

In infants and pediatric patients with a BGT that inadvertently comes out, traumatically or accidentally, before the tract is considered established, does confirming placement of the gastric replacement tube via the use of US versus a radiologic contrast study result in fewer negative outcomes?

*Recommendation*: In infants and pediatric patients with accidental or traumatic BGT dislodgement that occurs prior to when the tract is considered mature, we suggest using US, which is an emerging confirmation technique that may be equivalent to a contrast study for confirming BGT position. If institutionally feasible, US has the advantage of minimizing radiation. If US is not conclusive, a contrast study should be performed.
*Certainty of evidence*: Very Low
*Strength of recommendation*: Weak
*Delphi panel agreement*: 100%
*Validation panel agreement*: 100%

##### Descriptive

One study in 50 children who presented to the ED for dislodged BGT met the inclusion criteria for this question (Table 2).^23^ This was a prospective validation study supporting US as a technique with good agreement to radiologic contrast study. Comparing US to radiologic contrast confirmation demonstrated 96% sensitivity and 100% specificity for correct BGT placement confirmation. The investigators touted no radiation exposure and possible decreased ED LOS as benefits of US over radiologic contrast studies in this patient population (Table S1). Of the study subjects, all but one were <17 years old and that subject was 18.7 years old. (Personal communication with author, January 22, 2026.) This study is a true representation of patients who access the pediatric ED for BGT replacement issues as their BGT may have been placed at that facility when the subject was a minor and the care may still be provided by that facility as the person enters young adulthood. The bias rating for this study was “Low Risk” for the primary outcomes of interest (Table S2).

**Table 2:** Data summary for PICOT Question 13.

Reference	Population	Comparison of intervention versus comparator (*n*)	Age (SD)	Outcome: tube in place	Outcome: time to perform study (min)	Comments (including outcomes not found)
Frank et al.^23^	Pediatric patients aged 0–21 (all but one were <18) years who presented to the Peds ED with a dislodged GT who, after GT replacement, required confirmatory imaging were eligible for enrollment in the study. Criteria for confirmatory imaging included surgical GT placement <90 days from Peds presentation, stoma dilation, traumatic removal/replacement, or uncertainty about proper GT placement.	US was performed by pediatric US technicians (sonographers) following the GT US research protocol. Sonographers were asked to document any complications during US as well as their own certainty of correct GT placement. Subjects then proceeded to fluoroscopy for contrast injection of GT. Data were analyzed to determine the sensitivity and specificity of US, as compared with the contrast injection, with regard to determining proper placement of the tube in the stomach (*n* = 50).	47 months (54.89) Patients served as their own control	Control (radiologic contrast study): GT in place = 47 (94%) Not in place or uncertain = 3 (6%) Intervention (US): GT in place = 47 (94%) Not in place or not performed = 3 (6%) US had 96% sensitivity and 100% specificity	Control: *n* = 48 3.62 (3.66) Intervention: *n* = 50 5.78 (4.52) *P* = 0.0072	This was a validation study looking at 50 children who received US, with 48 proceeding to contrast study. Time for study procedure was statistically significantly longer but not clinically longer. This time variable does not include time in the ED waiting to have study done.

Abbreviations: ED, emergency department; GT, gastrostomy tube.

##### Rationale and discussion

A radiologic contrast study is the gold standard for tube placement verification but exposes the patient to radiation. This represents a harm, making radiologic contrast study something that should only be used when necessary. US is safe and effective and has been shown in at least one study to have strong concordance with radiologic contrast for confirming tube placement. This balance of benefits and harms forms the basis for the current recommendation.

Again, our recommendation is contingent on the definition of “feasible.” For the purpose of using US to confirm replacement BGT position, “feasible” means that the institution has the trained staff with proficiency in using US in this manner. Until more research on POCUS for this procedure is published, we suggest US be done in the radiology department with involvement of a pediatric radiologist.

It is important to note that in the study that met our inclusion criteria no data were presented comparing costs of both procedures.^23^ The average time spent doing US was 5.78 min, compared with a mean of 3.62 min for the contrast study.^23^ Of note, the US procedure involved instillation of normal saline or an oral electrolyte solution into the replacement BGT to enhance visualization in the stomach versus peritoneum. This study is promising, especially for institutions that have expertise in using POCUS for this clinical scenario. In order to be widely beneficial, it requires the availability of trained staff during all hours. At this time, we consider this study to be a promising, emerging technology that warrants further study and consideration. An important caveat to POCUS is that a radiologic contrast study should remain the gold standard for confirmation of replacement BGT, and any questionable results from POCUS should prompt a contrast study to be performed.

The use of US in clinical practice is growing because it is less invasive. One prospective study that did not meet our inclusion criteria as it looked at routine BGT and gastrojejunal tube replacement compared POCUS to radiologic contrast study for confirming replaced BGT.^21^ For gastric confirmation, the investigators also documented pH measurement, as the institution uses pH to confirm nasogastric tube placement. In 30 subjects, no gastric aspirate could be obtained; however, the average pH did indicate gastric acidity, with a mean pH of 4.1.^21^ Using POCUS, the balloon was visualized in the stomach in all but one subject, in whom it could not be identified. A comparison of the results for indeterminate or displaced tubes using POCUS (*n* = 16) versus radiologic contrast study (*n* = 4) showed the latter test to be superior.^21^

##### Future research

Research is needed that informs when using US might be comparable or superior to radiologic contrast studies. While a randomized controlled trial may not be practical, the use of an interrupted time series quasi‐experimental design would allow different hospitals to study the impact of US versus POCUS versus radiologic contrast and compare outcomes. Patient outcome measurements should include tube misplacements, LOS in the ED, cost of the interventions, and radiation exposure.

#### Question 14

In infants and pediatric patients with a BGT that comes out traumatically or accidentally before the tract is considered mature, does the use of a care bundle by staff compared to non‐use of a care bundle result in fewer negative outcomes?

*Recommendation*: In infants and children with a BGT that comes out accidentally or traumatically before the tract is considered mature, we recommend the use of a care bundle to standardize patient management and caregiver education.
*Certainty of evidence*: Very Low/Expert Opinion
*Strength of recommendation*: Strong
*Delphi panel agreement*: 100%
*Validation panel agreement*: 100%

One quasi‐experimental pre‐post policy implementation study met the inclusion criteria for this question (Table 3).^41^ The study did not find a significant difference between groups, but the number of patients that had a dislodgement in each group was low (17 pre, 22 post) and may have been underpowered to detect an effect. Children with a dislodged BGT that cannot be replaced at home will often present to the ED for medical help with replacement. The quasi‐experimental study by Osuchukwu et al. compared resource utilization and patient outcomes pre and post BGT algorithm implementation in the ED.^41^ The report included an algorithm that took into consideration clinical history, examination of the patient, length of time the BGT has been in place, and whether dilation was needed for replacement. The algorithm also included a recommendation to feed or flush through the newly replaced BGT to assess patient tolerance prior to discharge from the ED, along with indications for further imaging to confirm tube position, such as abdominal pain and vomiting post replacement.^41^ Before algorithm implementation, 17 subjects had a BGT dislodgement prior to 8 weeks post placement, compared to 22 children post algorithm implementation. There was no difference in ED LOS, use of radiologic contrast studies, or proportion of ED staff versus surgeon replacements. Four children were admitted post algorithm implementation, and all four required reoperation. Of the contrast studies done in the subjects with premature BGT displacement, 1.2% (*n* = 1) were malpositioned into the peritoneum. While this BGT was never used, another two subjects with BGT replacement outside of the hospital (home and daycare center) presented to the ED with malpositioned BGTs and peritonitis. The duration for which those misplaced BGTs had been in place was not reported. This study pointed out the clinical benefits of an algorithm in this clinical scenario, but also acknowledged that the algorithm may have increased resource utilization as it directed more patients toward a surgical consult. The bias rating for this study was “Serious Risk” (Tables S3 and S4).

**Table 3:** Data summary for PICOT Question 14.

Reference	Population/age (median [IQR])	Comparison of intervention versus comparator (*n*)	Age (SD)	Outcome: inappropriate additional radiation	Outcome: ED patient complications requiring additional interventions
Osuchukwu et al.^41^	Children ≤18 years old with dislodged BGT who presented to the ED <8 weeks post GT placement compared to those >8 weeks post placement Median age = 24 months [12, 60]	Implementation of an evidence‐based care algorithm for all patients who present to ED with GT dislodgement compared with children seen prior to implementation of the algorithm Pre‐implementation = 200; post‐implementation = 233	Median: 24 months (12, 60)	No significant difference between groups (pre and post implementation (125 pre group, 162 post group, *P* = 0.12)	Need for additional surgery: no significant difference between groups (pre and post implementation, but the numbers were small (1 pre group, 5 post group; *P* = 0.22)

Abbreviations: BGT, balloon gastrostomy tube; ED, emergency department; GT, gastrostomy tube; IQR, interquartile range.

##### Rationale and discussion

The use of a care bundle after traumatic or accidental dislodgement has several potential benefits. Theoretically, it may reduce the risk of misplacement, prevent delays in management, and assist in preserving the tract. Improving caregiver understanding may result in reduced tube dislodgements and emergency visits. While the findings from the one small study, likely underpowered, that directly met our inclusion criteria were null, findings extrapolated from research that did not meet our inclusion criteria corroborate the idea that a care bundle would be effective. The current recommendation is based on this, and that there are no known significant harms associated with providing a care bundle.

The care bundle content should be evidence informed and could include the following components:

- What to do if the BGT becomes dislodged at home prior to when the tract is considered mature
  a. Provide a new tube or device to stent the stoma and instruct caregivers to place it in the stoma to prevent spontaneous closure
  b. Secure the newly placed tube or stenting device with tape and do not access it
  c. Proceed to the ED immediately to confirm correct tube position

- Anticipatory guidance for the caregiver
  a. What to expect with BGT management
  b. How to handle routine, urgent, and emergent situations
  c. Who to contact in case of a problem, day or night

- ED staff assessment of the clinical situation and BGT stoma
  a. Include replacement verification methods recommended for various patient presentation scenarios

- Use of an algorithm to guide decision making
- 24/7 availability of staff to troubleshoot BGT problems remotely by phone or telehealth to avert an ED visit

The use of a care bundle that standardizes BGT‐related management and discharge education decreases downstream healthcare resource utilization. Studies that did not meet our inclusion criteria because they did not focus on inadvertent BGT dislodgement reported improved patient outcomes and/or decreased resource utilization comparing pre‐care and post‐care bundle implementation.^30^, ^31^, ^32^, ^44^ A quasi‐experimental study documented that children with a BGT whose caregiver participated in the pre‐procedure education class had significantly fewer unplanned ED or acute care visits (*n* = 288) compared with the group who had not attended the class (*n* = 498, *P* < 0.01).^31^ There was also a significantly shorter post‐placement LOS in the group that received the education, which the investigators surmised was related to the need for less time for families to become comfortable with BGT care.^31^ A similar study documented the benefits of a caregiver education program, including hands‐on training with a doll and written materials, documented post‐implementation office and ED visits decreased from 20 pre to 8 post, and from 26 pre to 11 post, respectively.^32^ One‐year outcomes were only statistically significant for decreased office visits (*P* < 0.001), but ED visits, readmissions, and phone calls were all lower post implementation.^32^ Both of these reports validate the benefits of an education‐focused care bundle prior to discharge from initial BGT insertion. A recently published study examining the use of a care bundle to mitigate complication differences in children with a newly placed BGT in high versus low socioeconomic living conditions reported similar outcomes in 90‐day post‐BGT placement dislodgements following bundle implementation.^44^ Moreover, this effect was sustained for 3 years.

Care bundles with a more comprehensive focus on initial BGT placement, patient evaluation, and management have documented clinical benefits that we believe can be extrapolated to this question despite not meeting our inclusion criteria. One quasi‐experimental study on hospital resource utilization compared pre and post implementation of a standard clinical pathway for BGT management that included pre‐procedure workup, identification of a medical home, nutrition evaluation with a discharge plan identified, determination of home health needs and supplies, and an evaluation of the overall family psychosocial situation.^30^ Subjects who were not part of the standard discharge instruction program required 58% additional hospital resources compared to 42% additional resources used by subjects who had the instruction program.^30^ Another such study documented a significant decrease in 90‐day dislodgement with a 47% risk reduction after implementation of a care bundle that focused on a standard pre‐procedure patient evaluation, intra‐procedure techniques, and post‐procedure education.^29^ These investigators performed process audits 1 year post care bundle implementation and found ≥75% compliance for all aspects of the care bundle. Accessing the healthcare system for assistance with a BGT replacement or management is often disruptive to a family, so decreased utilization of healthcare resources equates to less family life disruption. We believe that effective patient management of BGTs that become displaced before the tract is mature starts with an organized predischarge care bundle that includes caregiver education and a systematic approach to patient management in the ED.

##### Future research

Future studies should compare patient outcomes before and after implementation of a care bundle in the context of BGT dislodgement or for patients who require replacement but have an immature tract. Quasi‐experimental studies, such as large studies from different institutions, could compare patient outcomes pre and post implementation of care bundles that are more comprehensive versus focused on BGT dislodgement and replacement to discern which approach is most effective. Patient outcomes should include BGT dislodgement prior to track maturity, replacement difficulty, need for dilation, complications (such as bleeding or false tract formation), and methods used to confirm placement.

#### Question 15

In infants and pediatric patients with a BGT that accidentally or traumatically comes out before the tract is considered mature, does the use of a formal clinician education program focused on BGT replacement confirmation versus no education program result in fewer negative outcomes?

*Recommendation*: In infants and pediatric patients with a BGT that becomes dislodged accidentally or traumatically before the tract is considered mature, we recommend the development and implementation of a formal education program for clinicians who replace and manage these tubes.
*Certainty of evidence*: Very Low/Expert Opinion
*Strength of recommendation*: Strong
*Delphi panel agreement*: 100%
*Validation panel agreement*: 100%

One quasi‐experimental pre‐post design met our inclusion criteria and found a formal clinician education program to be beneficial by addressing inconsistent practice in the ED regarding the management of children who presented with a dislodged BGT (Table 4).^9^ The authors designed the study based on inconsistencies in methods used to confirm replacement BGT position with overuse of radiologic contrast studies, inadequate documentation, and infrequent referrals to a clinic for further management. They developed an algorithm to address these concerns, and staff were then educated on the use of the decision aide to guide practice with the intent to improve patient care. Components of the algorithm included provider assessment of the BGT, including review of initial placement procedure and pertinent history, physical examination with comprehensive health history, hydration status and medication review, determination of tract maturity with subsequent actions based on whether stomal dilation was needed, replacement of BGT, recommended confirmation method, and documentation.^9^ Education was provided in nine staff huddles and one quality improvement meeting by a pediatric surgery nurse practitioner. Of note, an evaluation of the education program by 22 participants indicated that 86% had changed practice based on the use of the algorithm, and 96% reported a better understanding of the entire process from assessment to replacement, confirmation, and documentation of the newly placed BGT. In terms of patient outcomes, the intervention decreased the use of radiologic contrast studies, thus decreasing additional radiation to the patients and decreased additional ED visits with displaced feeding tubes. The intervention did not decrease ED LOS. The bias rating for this study was "Serious Risk" (Tables S3 and S5).

**Table 4:** Data summary for PICOT Question 15.

Reference	Population/age (median)	Comparison of intervention versus comparator (*n*)	Age	Outcome: inappropriate additional radiation	Outcome: ED patient complications requiring additional interventions	Outcome: clinician knowledge score (SD)	Outcome: LOS in ED in patients with contrast study	Comments
Weszelits et al.^9^	Two populations, clinicians in Peds ED *n* = 26, patients who returned to ED with displaced GTs *n* = 53 Age not reported	Care of the child with displaced GT in the ED Algorithm and clinician education versus clinician knowledge (same data as intervention but during the pre‐implementation period) Measuring clinician knowledge and patient outcomes *n* = 22 clinicians *n* = 53 patients	Not reported	Control *n* = 9 Events 5 (56%) No Events 4 (44%) Intervention: *n* = 4 Events 1 (25%) No Events 3 (75%) *P* not reported	Readmission with displaced tube: control *n* = 34 Events 4 (12%) No events 30 (88%) Intervention: *n* = 19 Events 1 (5%) No events 18 (95%)	Pre‐test: *n* = 26 clinicians 80.45 (10.45) Post‐test *n* = 22 clinicians 90.91 (8.11) *P* = 0.001	Control *n* = 9 108 min Intervention: *n* = 4 148 min *P* not reported	Intervention increased clinician knowledge, decreased inappropriate contrast studies, decreased return to ED with displaced tubes, but did not decrease ED LOS

Abbreviations: ED, emergency department; GT, gastrostomy tube; IQR, interquartile range; LOS, length of stay.

##### Rationale and discussion

A formal clinician education program for managing BGT dislodgement prior to tract maturation may be key to reducing clinician error, maintaining consistency to care between providers, and ensuring safe replacement practices. Inadequately trained clinicians have the potential to cause harm by failing to recognize situations where there is a high risk of false tract formation during BGT replacement or by overutilizing radiologic contrast studies. While such a program involves a time investment and use of institutional resources, these are minor and are outweighed by the benefits for this population.

The formal education program should be institution specific and include, at a minimum:

- A didactic course on routine and non‐routine BGT replacement covering:
  o Pertinent clinical history and assessment of the patient
  o Assessment of the stoma, including how long the BGT has been out of the tract and measures at home (if any) to replace the BGT
  o Assessment of the type and size of the BGT, with consideration of resizing if indicated
  o How to determine BGT replacement needs to be escalated to more senior staff
  o Routine methods and escalation strategies to verify BGT replacement position

- Development of an algorithm that delineates who (by staff category) can replace BGT and how the BGT is assessed and managed, including:
  o BGT and patient assessment as described above
  o Defining who can perform routine versus nonroutine replacements
  o Extenuating circumstances when increased vigilance should be used, such as bleeding, comorbid conditions that could delay tract maturation, or dislodgement of a newly placed BGT
  o Who can dilate a BGT
  o When a surgery consult is indicated
  o Replacement BGT confirmation methods and when to escalate
  o Post‐replacement monitoring before discharge
  o Documentation

- Competency validation:
  o Number of required initial supervised BGT replacements by a senior staff member who is authorized to be a trainer
  o Number of annual BGT replacements required to maintain ongoing competency

- Components of procedure documentation:
  o Development of a standard template for the BGT replacement procedure that can be part of the patient's electronic health record, including:
    o Initial assessment information
    o Difficulty in replacement, including need to dilate the stoma
    o Include type, size, and brand of BGT used
    o Amount of water placed in the balloon
    o Patient tolerance
    o Evidence of bleeding post replacement
    o Method(s) used to confirm replacement BGT placement
    o Text box to document other pertinent information not in the template

The study by Weszelits et al. and others previously cited^2^, ^3^ tout the wisdom of using an interdisciplinary group of key stakeholders to develop a staff education program based on a care bundle or algorithm to standardize the process of BGT displacement management.^29^, ^37^ Stakeholders included physicians, physician assistants, and advanced practice nurses as staff who replaced dislodged BGT, and VanDerheof's education program was designed to allow registered nurses to perform routine BGT replacements in the ED.^9^, ^37^ Increasingly, nurses are assuming more responsibility for all aspects of BGT management. As with any quality improvement process, we recommend periodic evaluation of patient outcomes and staff knowledge to monitor adherence to the processes taught to staff.

##### Future research

Randomized controlled trials may not be feasible or ethical to address this question, but larger studies comparing patient outcomes or resource utilization pre and post staff education programs would validate the need to make this best practice for BGT replacement when the tract is not mature. Future studies should evaluate the effectiveness of formal clinician education. While randomizing one group to no clinician education would not be ethical, other observational and quasi‐experimental designs might be pursued. This could include stepped‐wedge studies, quasi‐experimental cluster‐controlled trials, or quasi‐experimental retrospective designs examining our population at a hospital pre‐policy and post‐policy change. Research that considers which components of education are most effective should also be considered. Relevant outcomes for these studies include BGT dislodgement rates, infection, complications related to misplacement, and staff confidence and competence at BGT replacement. Research on how this clinician education is done—that is, online versus in‐person classes or using a simulation lab—would guide institutions to efficiently and effectively educate staff.

## CONCLUSION

The use of BGTs to provide nutrition to infants and children has increased substantially over the years and now involves surgeons, interventional radiologists, pediatric gastroenterologists, ED physicians, advanced practice providers, experienced staff nurses, and caregivers. This variety of specialties makes a consistent approach to BGT management even more vital. Standardizing both routine confirmation methods and nonroutine evaluation and replacement procedures is therefore essential to maintaining safe and appropriate care. While little data exist that can directly comment on the effect of these practices on patient care, the recommendations in this guideline represent the opinions of a highly experienced team and utilize sound academic logic, which is also corroborated by observational studies in the field. High‐quality prospective research using the same variables is lacking and prompts a call to action. Randomized control and/or quasi‐experimental studies will be needed to provide a more robust evidence base for these important questions.

## AUTHOR CONTRIBUTIONS

**Beth Lyman**: Conceptualization; Writing—original draft; Writing—review and editing; Data curation. **Loren Berman**: Data curation; Writing—review and editing; Conceptualization. **Kathleen Carr**: Data curation; Writing—review and editing; Conceptualization. **Cailin Frank**: Data curation; Writing—review and editing; Conceptualization. **Peggi Guenter**: Data curation; Writing—review and editing; Conceptualization; Writing—original draft; Project administration. **Rachel Kassel**: Writing—review and editing; Data curation; Conceptualization. **Janet Kimble**: Conceptualization; Writing—review and editing; Data curation. **Carol McGinnis**: Conceptualization; Writing—review and editing; Data curation. **Silvana Oppedisano**: Conceptualization; Writing—review and editing; Data curation. **Elizabeth A. Paton**: Conceptualization; Writing—review and editing; Data curation. **Gina Rempel**: Conceptualization; Writing—review and editing; Data curation. **Derek S. Wakeman**: Conceptualization; Writing—review and editing; Data curation. **Jacob T. Mey**: Data curation; Writing—review and editing; Formal analysis. **Sarah Peterson**: Data curation; Writing—review and editing; Formal analysis. **Liam McKeever**: Formal analysis; Writing—review and editing; Writing—original draft; Conceptualization; Methodology; Supervision; Data curation; Project administration.

## CONFLICT OF INTEREST STATEMENT

Beth Lyman is a consultant for Avanos, Cardinal Health, and unpaid advisor for Otsuka Pharmaceuticals on issues unrelated to the current project. The remaining authors declare no conflicts of interest.

## Supporting information

Supplemental Appendix.
