# Supplementary material for: Guidelines for replacement of a balloon gastrostomy tube in infants and pediatric patients: The American Society for Parenteral and Enteral Nutrition
Source: JPEN J Parenter Enteral Nutr. 2026 Mar 26;50(4):462–85. doi: 10.1002/jpen.70080 (PMC13169229; doi:10.1002/jpen.70080)
Supplement: Supplementary file 1 — Supplemental Appendix. [file JPEN-50-462-s001.docx]

**Supplemental Appendix**

***Table of Contents***

**Table S1: Question 13 Summary of Evidence**

Page 2

**Table S2: Results of the QUADAS II Bias Tool for Question 13**

Page 3

**Table S3: Results of the ROBINS-I Bias Tool for Questions14 & 15**

Page 3

**Table S4: Question 14 Summary of Evidence**

Page 4

**Table S5: Question 15 Summary of Evidence**

Page 5-6

**Table S1: Question 13 Summary of Evidence**

| **Outcomes** | **Number of participants (studies) Follow-up** | **Certainty of the evidence (GRADE)** | **Impact** |
| --- | --- | --- | --- |
|  |  |  |  |
| Tube in Place | (1 non-randomized study) | ⨁◯◯◯ Very low^a^ | Control (Radiologic contrast study):  G-tube in place=47(94%)  Not in place or uncertain= 3 (6%)  Intervention (Ultrasound):  G-tube in place=47(94%)  Not in place or not performed= 3 (6%)  US had 96% sensitivity and 100% specificity |
| Time to Perform Study (min) | (1 non-randomized study) | ⨁◯◯◯ Very low^a^ | Control: n=48  3.62 (3.66)  Intervention:n=50  5.78(4.52)  P= 0.0072  Patients served as their own controls. |

#### Explanations

a. This was based on one small validation study in 55 patients.

**Table S2: Results of the QUADAS II Bias Tool for Question 13**


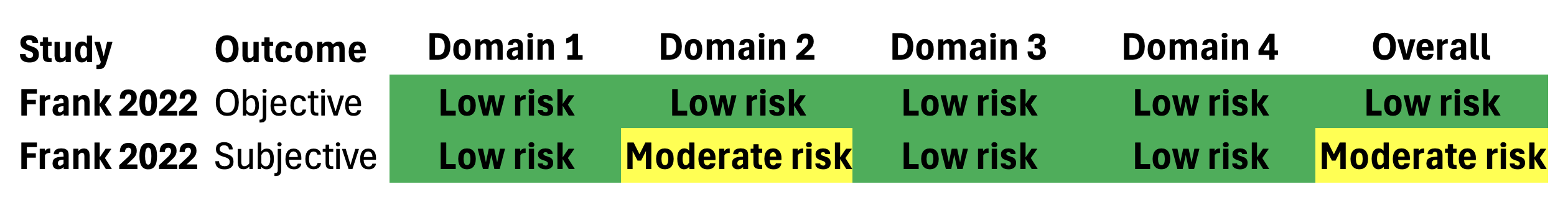


**Table S3: Results of the ROBINS-I Bias Tool for Questions14 & 15**


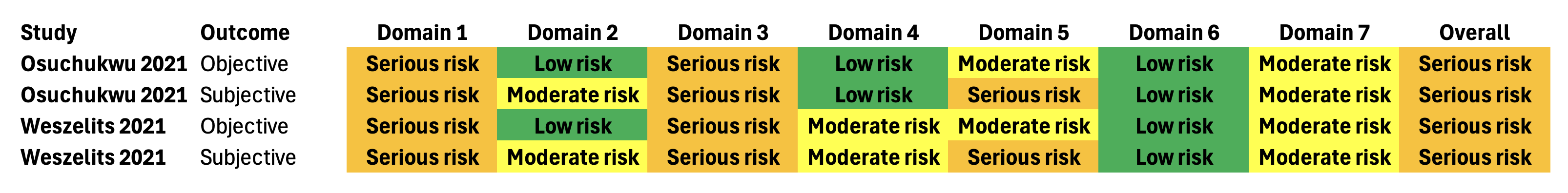


**Table S4: Question 14 Summary of Evidence**

| **Outcomes** | **Number of participants (studies) Follow-up** | **Certainty of the evidence (GRADE)** | **Impact** |
| --- | --- | --- | --- |
|  |  |  |  |
| Inappropriate Additional Radiation | (1 non-randomized study) | ⨁◯◯◯ Very low^a,b^ | No significant difference between groups (pre- and post-implementation, (125 Pre-group, 162 Post-group, p=0.12). |
| ED Patient Complications Requiring Additional interventions | (1 non-randomized study) | ⨁◯◯◯ Very low^a,b^ | **Need for Additional Surgery:**  No significant difference between groups (pre- and post-implementation, but the numbers were small (1 Pre-group, 5 Post-group, p=0.22). |

#### Explanations

a. Non-randomized

b. No adjustment for key confounders

**Table S5: Question 15 Summary of Evidence**

| **Outcomes** | **Number of participants (studies) Follow-up** | **Certainty of the evidence (GRADE)** | **Impact** |
| --- | --- | --- | --- |
|  |  |  |  |
| Clinician Knowledge Score | (1 non-randomized study) | ⨁◯◯◯ Very low^a^ | Pretest: n=26 clinicians  80.45 (10.45)  Post-test n=22 clinicians  90.91 (8.11)  p=0.001 |
| Inappropriate Additional Radiation | (1 non-randomized study) | ⨁◯◯◯ Very low^a^ | Control n=9  Events 5 (56%)  No Events 4 (44%)  Intervention: n=4  Events 1 (25%)  No Events 3 (75%)  P=not reported |
| LOS in ED in patients with contrast study | (1 non-randomized study) | ⨁◯◯◯ Very low^a^ | Control n=9  108 min  Intervention: n=4  148 min  P=not reported |
| ED readmission with displaced tube | (1 non-randomized study) | ⨁◯◯◯ Very low^a^ | Control n=34  Events 4 (12%)  No Events 30 (88%)  Intervention: n=19  Events 1 (5%)  No Events 18 (95%) |

#### Explanations

a. This is based on a single quasi-experimental study in 53 patients
